# The value of anti-Mullerian hormone for diagnosing polycystic ovary syndrome in adolescents: a case-control study based on an epidemiological survey

**DOI:** 10.3389/frph.2026.1907699

**Published:** 2026-08-04

**Authors:** Yu Hong, Enping Li, Dongzi Yang, Zehong Zhou, Zhe Dong

**Affiliations:** 1 Department of Obstetrics and Gynecology, Memorial Hospital of Sun Yat-Sen University, Guangzhou, China; 2 Department of Obstetrics and Gynecology, Kiangwu Hospital, Macao SAR, China

**Keywords:** adolescence, anti-Müllerian hormone, auxiliary diagnostic biomarker, polycystic ovary syndrome, polyendocrine metabolic ovarian syndrome

## Abstract

**Background:**

Updated 2023 international evidence-based guidelines do not recommend anti-Müllerian hormone (AMH) for diagnosing adolescent polycystic ovary syndrome (PCOS, also newly termed polyendocrine metabolic ovarian syndrome [PMOS]) due to limited specificity. Physiological multifollicular ovaries are prevalent in teenagers, and existing studies show massive heterogeneity in participant age, BMI, AMH detection platforms and cut-off thresholds without unified adolescent-specific standards. This study aimed to analyze serum AMH correlations with core adolescent PCOS phenotypes and evaluate the auxiliary diagnostic performance of AMH for adolescent PCOS.

**Methods:**

Based on a cross-sectional school-based epidemiological survey among adolescent girls aged 14–18 years, we enrolled 211 healthy girls, 158 girls with oligo-/amenorrhea, 38 girls with isolated clinical hirsutism, 51 girls with isolated biochemical hyperandrogenemia, and 39 adolescent PCOS patients for case-control analysis. Pearson's correlation coefficients were calculated to determine the correlations between AMH and various parameters. Receiver Operating Characteristic (ROC) curve analysis was performed to examine the diagnostic performance of AMH.

**Results:**

Serum AMH concentrations were significantly higher in girls with oligo-/amenorrhea and isolated biochemical hyperandrogenemia relative to healthy controls (*P* < 0.05), while isolated hirsutism presented comparable AMH levels with controls (*P* > 0.05). Adolescent PCOS patients had the highest AMH levels among all subgroups (*P* < 0.05). AMH was positively correlated with all circulating androgen markers (T, FAI, A2, fT, DHEAS) but showed no correlation with age, menarcheal age, gynecological age, BMI, WHR or SHBG. Within the PCOS cohort, only serum testosterone maintained an independent positive correlation with AMH (r = 0.450, *P* = 0.004). ROC analysis showed an AUC of AMH for adolescent PCOS of 0.717 (95%CI: 0.625–0.809, *P* < 0.001). Bootstrap-validated optimal AMH cut-off value was 4.0 ng/mL, with sensitivity 51.3% (95CI: 35.4%–67.2%) and specificity 86.1% (95CI: 81.2%–90.0%). *post-hoc* power analysis demonstrated moderate statistical power of 0.74 for this ROC model due to limited PCOS sample size (*n* = 39).

**Conclusions:**

Serum AMH is closely correlated with two core diagnostic manifestations of adolescent PCOS: ovulatory dysfunction and biochemical hyperandrogenemia. Nevertheless, the low sensitivity of the 4.0 ng/mL threshold leads to nearly half missed PCOS cases if used alone for screening. Combined with inherent incorporation bias, absence of ovarian ultrasound data and consistent 2023 guideline recommendations, AMH can only serve as an adjunctive auxiliary biomarker rather than an independent diagnostic or screening tool for adolescent PCOS. Multicenter studies with standardized ovarian ultrasonography and external validation cohorts are required to establish generalizable age-specific AMH cut-offs for adolescents.

## 1 Introduction

Polycystic ovary syndrome (PCOS), recently renamed polyendocrine metabolic ovarian syndrome (PMOS) in updated consensus statements, is a common endocrine-metabolic disease with high prevalence among adolescent girls. Diagnosis in this population remains challenging owing to substantial overlap between physiological pubertal ovarian changes and pathological PCOM/PCOS phenotypes (1). Clinically, PCOS adolescents frequently suffer from oligomenorrhea or amenorrhea secondary to chronic anovulation, yet the exact pathogenic interaction between ovarian follicular dysfunction and hormone dysregulation remains incompletely clarified.

Anti-Müllerian hormone (AMH), a member of the transforming growth factor-beta superfamily, is exclusively secreted by granulosa cells of small pre-antral and early antral follicles. Patients with polycystic ovaries possess a markedly expanded pool of small growing follicles, leading to elevated circulating AMH. AMH restrains excessive primordial follicle activation and early follicle recruitment; its abnormal over-expression contributes to follicular arrest and anovulation. Serum AMH exhibits stable concentrations across the entire menstrual cycle, with high intra-individual measurement reproducibility superior to ultrasound antral follicle count (AFC) (2–4). For adult women, AMH is accepted as a reliable AFC surrogate to define polycystic ovarian morphology (PCOM). However, the 2023 International Evidence-Based Guidelines explicitly discourage AMH testing for adolescent PCOM/PCOS diagnosis, citing insufficient specificity in teenage populations (5).

Multiple observational studies have explored AMH-PCOS associations in adolescents, yet published results remain contradictory. Major sources of heterogeneity include variable participant age ranges, BMI stratification, distinct AMH immunoassay platforms and inconsistent diagnostic cut-off thresholds (6). Further targeted cohort analyses focusing on Asian late adolescents are urgently needed to develop population-specific reference values. Therefore, this population-based case-control study aimed to characterize AMH variations across distinct adolescent PCOS-related phenotypes and quantify the auxiliary diagnostic efficacy of serum AMH via ROC and multivariable regression modeling.

## 2 Materials and methods

### 2.1 Subjects

All participants were 14–18-year-old tenth-grade Chinese girl students recruited from a previously completed cross-sectional epidemiological survey. Five subgroups were defined according to clinical and biochemical phenotypes: PCOS group (*n* = 39), healthy control group (*n* = 211), isolated oligo-/amenorrhea group (O/A, *n* = 158), isolated clinical hirsutism group (*n* = 38), isolated biochemical hyperandrogenemia group (BH, *n* = 51).

#### 2.1.1 Diagnostic criteria for adolescent PCOS

PCOS diagnosis strictly followed the 2023 international adolescent PCOS guideline: (1) ovulatory dysfunction: ≤8 menstrual cycles annually, or cycle length <21 days/ > 35 days without recent hormonal intervention; (2) concurrent clinical hyperandrogenism [modified Ferriman-Gallwey (mFG) hirsutism score ≥6] and/or biochemical hyperandrogenemia (T ≥ 2.28 nmol/L, A2 ≥ 5.20 nmol/L, FAI ≥4.37); (3) secondary causes excluded via serum TSH, PRL and 17-OHP testing: thyroid dysfunction (TSH outside 0.35–5.5 mIU/L), hyperprolactinemia (PRL >24 μg/L), congenital adrenal hyperplasia (17-OHP >6 nmol/L).

#### 2.1.2 Healthy control inclusion criteria

① Post-menarche duration ≥2 years; ② regular menstrual cycles of 21–35 days with 3–7 days bleeding duration; ③ mFG hirsutism score <6; ④ normal serum androgen levels without biochemical hyperandrogenemia.

#### 2.1.3 PCOS-related isolated phenotype subgroup definitions

##### 2.1.3.1 O/A group

oligo-/amenorrhea without clinical or biochemical hyperandrogenemia;

##### 2.1.3.2 Isolated hirsutism group

regular menstruation, normal serum androgens, mFG ≥6;

##### 2.1.3.3 Isolated BH group

regular menstruation, normal mFG score, elevated circulating androgens

### 2.2 Clinical examination

All anthropometric measurements were conducted with participants in light indoor clothing without shoes. Height and weight were recorded by digital electronic scale to calculate body mass index (BMI, kg/m^2^). Waist circumference (narrowest abdominal level above umbilicus) and hip circumference (maximum gluteal level) were measured to compute waist–hip ratio (WHR). The modified Ferriman-Gallwey scoring system was applied to quantify hirsutism severity by trained gynecologists blinded to laboratory results.

### 2.3 Hormonal analysis

Fasting venous blood was collected after 8-hour overnight fasting. Plasma free testosterone (fT), sex hormone-binding globulin (SHBG), androstenedione (A2), dehydroepiandrosterone sulfate (DHEAS), 17-hydroxy-progesterone (17-OHP) and AMH were quantified by commercial ELISA kits. Serum T, PRL and TSH were detected via chemiluminescence immunoassay. Free androgen index (FAI) was calculated as [T (nmol/L)x100]/SHBG (nmol/L).

Serum AMH was measured using human AMH ELISA kit (Merck KGaA, Darmstadt, Germany, catalog No. RAB1936, detection range: 0.164–40 ng/mL). Intra-assay coefficient of variation (CV) < 10%, inter-assay CV <12%. All samples were tested in duplicate by two independent blinded laboratory technicians; mean duplicate values were adopted for statistical analysis following manufacturer standard operating protocols.

### 2.4 Statistical analysis

Statistical analyses were performed using SPSS 26.0 and G*Power 3.1 for *post-hoc* power calculation.

Normality assessment: Shapiro–Wilk test was used for all continuous variables. Normally distributed data were expressed as mean ± standard deviation (x¯ ± s); non-normal data were reported as median (interquartile range, IQR). Two-group comparisons for normal continuous variables adopted independent samples t-test; multi-group comparisons used one-way ANOVA with Tukey's *post-hoc* test and Bonferroni correction for pairwise contrasts. Non-normal continuous variables were analyzed by Kruskal–Wallis H test with Dunn–Bonferroni *post-hoc* correction. Categorical count data were presented as *n* (%) and compared via *χ*^2^ test or Fisher's exact test for small cell counts.

Correlation analysis: Pearson correlation for normally distributed paired variables; Spearman’s rank correlation for non-normal indicators including serum AMH.

Multiple linear regression (PCOS subgroup): Stepwise linear regression with AMH as dependent variable and all clinical/biochemical indices as independent predictors. Regression coefficient, overall model *P*-value and adjusted R^2^ were reported to evaluate model fitting quality.

Diagnostic efficiency analysis: Univariable ROC curve was plotted to calculate AUC, optimal cut-off, sensitivity, specificity and Youden's index with 95% CIs for diagnostic metrics. Bootstrap resampling (1,000 iterations) was implemented for internal validation of the 4.0 ng/mL cut-off to reduce overfitting risk. Multivariable binary logistic regression was constructed adjusting for age, gynecological age, BMI and serum testosterone to identify independent AMH predictive value for PCOS.

Post-hoc power analysis: Given n(case) = 39, n(control) = 211, *α*=0.05, observed AUC=0.717, the statistical power of ROC discrimination model was calculated as 0.74, indicating moderate stability with wide AUC confidence intervals. *P* < 0.05 was defined as statistically significant for all analyses.

## 3 Results

### 3.1 Serum AMH levels across all phenotypic subgroups

Table 1 presents both mean ± SD and median (IQR) of AMH, with Bonferroni-corrected pairwise *P*-values between subgroups. Compared with healthy controls, AMH was significantly elevated in O/A and isolated BH groups (*P* < 0.05). Isolated hirsutism showed no AMH difference versus controls (*P* > 0.05). PCOS girls exhibited significantly higher AMH than O/A girls (*P* < 0.05).

**Table 1:** Serum AMH levels in healthy girls and adolescents with distinct PCOS-related phenotypes.

Group	n	AMH (ng/mL) x¯ ± s	AMH (ng/mL) median(IQR)
Control	211	2.40 ± 1.76	2.02 (1.13–3.07)
O/A	158	3.2 ± 2.44^a^	2.75 (1.44–4.12)
Isolated hirsutism	38	2.52 ± 1.91	2.11 (1.20–3.23)
Isolated BH	51	3.7 ± 2.16^a^	3.38 (2.15–4.66)
PCOS group	39	4.62 ± 3.68^a^^,^^b^	4.10 (2.48–5.93)

O/A = oligo/amenorrhea; BH = biochemical hyperandrogenism.

a vs Control, *P* < 0.05 (Bonferroni corrected).

b vs O/A group, *P* < 0.05 (Bonferroni corrected).

### 3.2 Serum AMH levels across all phenotypic subgroups-correlation between serum AMH and clinical/biochemical indicators

Shapiro–Wilk test confirmed AMH followed non-normal distribution; thus Spearman's rank correlation was adopted for all correlation analyses. In the total pooled cohort, AMH had significant positive correlations with all androgen markers (FAI, T, A2, fT, DHEAS, all *P* < 0.05) and no correlation with age, menarcheal age, gynecological age, BMI, WHR, mFG score or SHBG (all *P* > 0.05, Table 2). Within the PCOS subgroup, only testosterone maintained a significant positive association with AMH (r = 0.450, *P* = 0.004). Stepwise linear regression generated the predictive equation y = 2.693 + 0.471 × 1 (x1 = T), overall model *P* = 0.002, adjusted R^2^ = 0.186, demonstrating testosterone explained 18.6% of AMH variance in PCOS adolescents.

**Table 2:** Spearman's correlation between AMH and clinical/biochemical indicators (total cohort).

Index	Spearman *r*	*P*
Age	−0.047	0.350
Menarche age	−0.039	0.430
Years after menarche	0.008	0.867
BMI	0.030	0.553
WHR	−0.027	0.584
F-G score	0.031	0.531
FAI	0.207	**0** **.** 000*
T	0.309	**0** **.** 000*
A2	0.159	**0** **.** 001*
fT	0.105	**0** **.** 035*
DHEAS	0.120	**0** **.** 016*
SHBG	0.040	0.428

BMI, body mass index; WHR, waist–hip ratio; FAI, free androgen index; T, testosterone; A2, androstenedione; fT, free testosterone; DHEAS, dehydroepiandrosterone sulfate; SHBG, sex hormone binding globulin.

Bold values represent statistical significance.

*p < 0.05.

### 3.3 ROC diagnostic performance of AMH for adolescent PCOS

**Figure 1:**
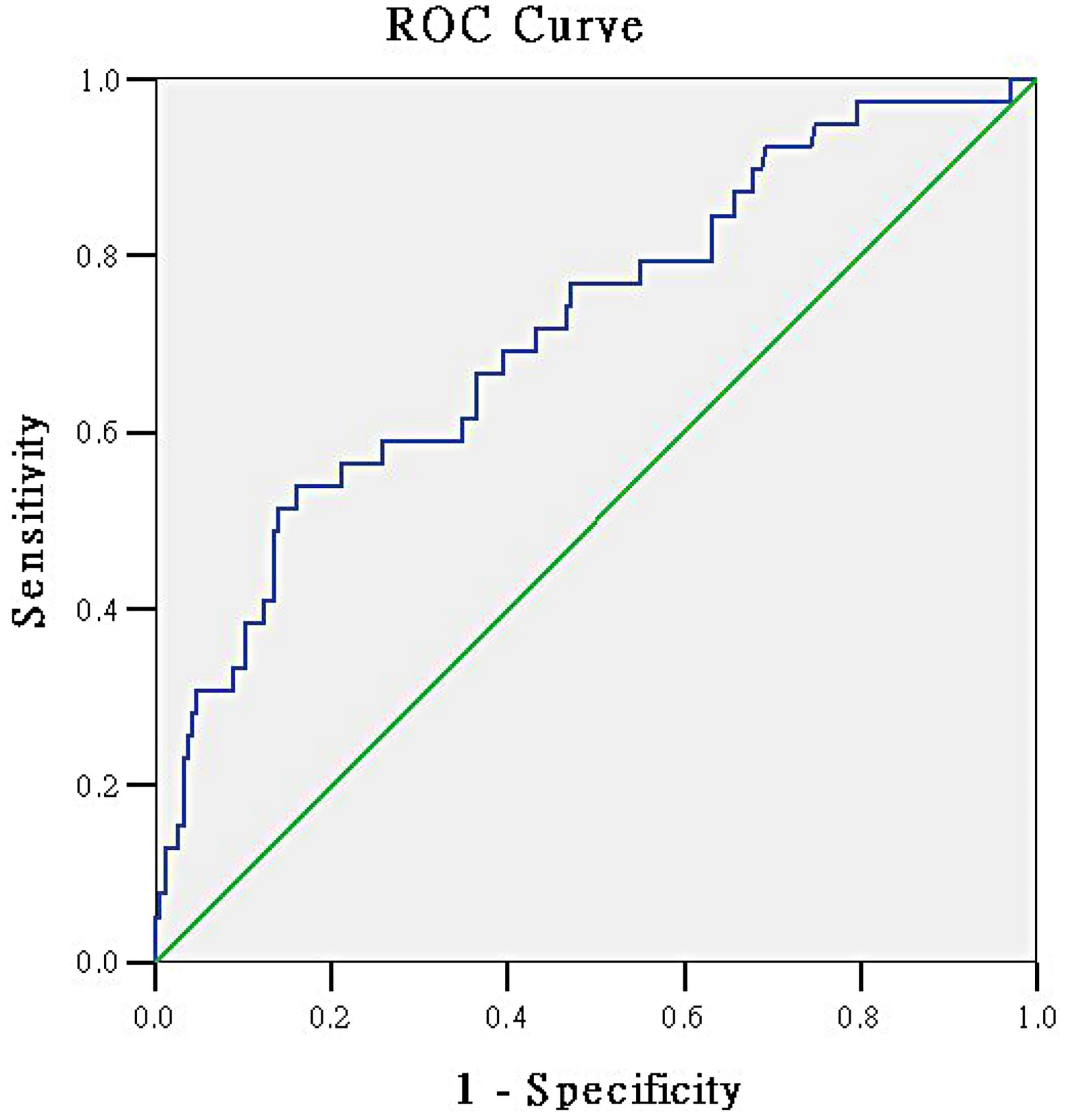
AUC in ROC analysis of serum AMH for diagnosing adolescent PCOS.

As shown in Table 3. and Figure 1., ROC analysis revealed that the area under the curve (AUC) for serum AMH in diagnosing adolescent PCOS was 0.717 (*p* < 0.001).

**Table 3:** AUC and 95% CI of AMH for discriminating PCOS from healthy adolescents.

	AUC	Standard error	*p* value	95% confidence interval
				Lower limit Upper limit
Adolescent PCOS	0.717	0.047	0.000	0.625 0.809

Bootstrap internal validation confirmed 4.0 ng/mL as the optimal threshold, yet the low sensitivity (51.3%) indicated nearly half PCOS cases would be misclassified as negative when relying solely on AMH testing (shown in Table 4). Multivariable logistic regression showed AMH remained a weak independent predictor of PCOS after adjusting for age, gynecological age, BMI and testosterone (OR=1.18, 95%CI:1.03–1.35, *P* = 0.015).

**Table 4:** Diagnostic metrics at sequential AMH cut-off values (with 95% CI for sensitivity/specificity).

Cutoff value for diagnosis (ng/mL)	Sensitivity (95%CI)	Specificity (95%CI)	Youden index
2.5	66.7 (50.2–80.1)	63.4 (56.6–69.7)	0.301
3.0	59.0 (42.8–73.7)	71.4 (64.9–77.2)	0.304
3.5	53.8 (37.9–69.2)	81.8 (76.0–86.6)	0.356
**4.0**	**51.3** **(35.4–67.2)**	**86.1** **(81.2–90.0)**	**0** **.** **374**
4.5	38.5 (23.7–55.1)	89.3 (84.9–92.7)	0.278

The bold values ​​in the table indicate the optimal threshold.

## 4 Discussion

Oligomenorrhea and amenorrhea are extremely prevalent among adolescent girls, with 16.6%–23.1% prevalence reported in Asian epidemiological surveys including our local Chinese cohort and Nepalese teenage populations (7, 8). Pubertal hypothalamic-pituitary-ovarian (HPO) axis immaturity fails to form estrogen positive feedback signaling, leading to transient anovulation within 3 years post-menarche (9). Transvaginal ultrasound is the traditional gold standard to distinguish anovulatory etiologies, yet serum AMH offers a non-invasive alternative screening biomarker without pelvic examination discomfort for adolescents. Patients with premature ovarian insufficiency display reduced AMH, while central hypogonadotropic hypogonadism or hyperprolactinemia present normal AMH levels; only PCOS-related multifollicular ovaries drive sustained AMH elevation (10–12).

In this study, adolescents with oligo-/amenorrhea had significantly higher AMH than healthy controls, and PCOS patients demonstrated the highest AMH concentrations. Transient pubertal anovulation induces physiological multifollicular ovaries (MFO) with expanded small follicle pools and elevated granulosa cell AMH secretion, which resolves spontaneously once regular ovulation is established (13). Villarroel et al. previously confirmed elevated AMH in adolescents with PCOM independent of age and hyperandrogenism (14). The 2023 PCOS guideline permits AMH substitution for AFC/PCOM in adults but explicitly restricts its adolescent application, largely due to widespread physiological MFO in teenagers without standardized age-specific AMH cut-offs to separate physiological and pathological follicular excess (5). A major limitation of our study is the complete absence of ovarian ultrasound AFC/PCOM data, which prevents direct analysis of AMH-AFC correlation and eliminates our capacity to test whether AMH could act as a surrogate PCOM marker in adolescents. This critical data gap substantially weakens the interpretability of our ROC AUC results and is fully acknowledged in the dedicated limitations subsection below.

Notably, isolated clinical hirsutism (*n* = 38) exhibited no significant AMH elevation relative to healthy controls, a negative result requiring mechanistic and ethnic interpretation. First, the mFG ≥6 hirsutism threshold adopted in Western guidelines is overly strict for East Asian adolescents, who present milder terminal hair growth at baseline; this conservative cut-off may fail to capture mild peripheral hyperandrogenism without concurrent ovarian follicular overactivity. Second, serum AMH secretion originates exclusively from ovarian granulosa cells, whereas clinical hirsutism reflects peripheral hair follicle androgen sensitivity rather than intraovarian androgen excess. Circulating bioavailable androgens directly stimulate granulosa cell AMH overproduction, explaining why isolated biochemical hyperandrogenemia (not isolated hirsutism) drives significant AMH upregulation in our cohort.

Consistent with multiple prior cross-sectional analyses (15–17), our Spearman correlation confirmed AMH positive association with all circulating androgen biomarkers across the full adolescent cohort, and testosterone represented the sole independent hormonal correlate of AMH within the PCOS subgroup. The underlying regulator*y* axis is well-established: AMH increases GnRH pulse frequency at the hypothalamic level, stimulating excessive pituitary LH secretion. Elevated LH overactivates ovarian theca cells to synthesize surplus androgens, creating a bidirectional positive feedback loop between AMH and hyperandrogenism that sustains follicular arrest and chronic anovulation (18, 19). IVF cohorts further confirm higher AMH in hyperandrogenic PCOS versus normoandrogenic PCOS patients; anovulatory PCOS individuals carry greater theca-cell androgen synthetic capacity than ovulatory counterparts (20–22). Among irregularly menstruating adolescents, biochemical hyperandrogenemia coexists far more frequently than isolated clinical hirsutism (23), consistent with our AMH subgroup gradients.

Contradictory *in vitro* evidence exists regarding testosterone's direct regulatory effect on granulosa AMH expression. Crisosto's bovine granulosa cell experiment demonstrated T-mediated AMH downregulation at high follicular androgen concentrations (24), while Pellatt's human PCOS granulosa culture reported elevated AMH secretion (25). This discrepancy highlights context-dependent ovarian signaling differences between normal physiological follicles and dysregulated polycystic follicle pools unique to PCOS patients, warranting further primary human ovarian tissue validation.

Ultrasound AFC assessment carries substantial clinical limitations for adolescent populations: transvaginal scanning is frequently refused by sexually inexperienced teenagers, and AFC counting suffers marked inter-operator variability dependent on ultrasound equipment quality and sonographer experience (26, 27). Serum AMH testing overcomes these drawbacks with cycle-independent stability, high intra-assay reproducibility and minimal operator bias (28). Nevertheless, the 2023 international PCOS guideline and subsequent meta-analyses by Kim et al. conclude insufficient diagnostic accuracy to recommend AMH for adolescent PCOS screening, citing severe cross-study heterogeneity from variable age strata, BMI distribution and immunoassay platforms (6, 26). Our study's ROC AUC of 0.717 aligns with previously published adolescent AMH diagnostic meta-analysis pooled AUC values ranging 0.61–0.66 (28–31), yet our 4.0 ng/mL cut-off achieved higher specificity (86.1%) at the cost of low 51.3% sensitivity. The high false-negative rate means nearly half of true adolescent PCOS patients will be missed if AMH testing is used alone for population screening. The acceptable specificity only confers limited auxiliary exclusion value: elevated AMH alongside oligomenorrhea or biochemical hyperandrogenemia can increase clinical suspicion, while normal AMH cannot reliably rule out PCOS diagnosis.

An additional important interpretative limitation is incorporation bias in our ROC analysis design. Our PCOS diagnostic criteria require both ovulatory dysfunction and hyperandrogenism, two phenotypes intrinsically positively correlated with serum AMH as demonstrated in our correlation analyses. Using AMH to predict a composite outcome defined by its own correlated biomarkers artificially inflates AUC values and overestimates true standalone diagnostic performance. This bias further reinforces that AMH can not independently confirm adolescent PCOS and must be interpreted comprehensively alongside menstrual history, clinical hyperandrogenism signs and full androgen laboratory profiles.

Our previous research also conducted a preliminary exploration of this area including patients in late adolescence who had experienced menarche for more than three years. We found that the receiver operating characteristic (ROC) of AMH for diagnosing PCOS was only 0.664, with AMH 8 ng/mL as the optimal diagnostic cutoff value. Sensitivity and specificity were 61.7% and 70%, respectively (29). In the only meta-analysis so far specifically in adolescents, 11 included studies assessing the accuracy of AMH for the diagnosis of adolescent PCOS displayed pooled sensitivity and specificity of 0.66 and 0.78, respectively (30). Compared with patients in puberty, AMH has a relatively higher diagnostic value for patients in adulthood, but its diagnostic efficacy is still significantly lower than that reported by Pigny (31). In 2010, Australian scholar Hart (32) found in his study of adolescent populations (aged 14.5–17.6 years) that the ROC of AMH for diagnosing PCOS as defined by the National Institutes of Health (NIH) was 0.61, with the optimal diagnostic cutoff value showing sensitivity and specificity of only 52.8% and 66.1%, respectively. Our current study cohort was restricted to 14–18-year-old late pubertal tenth-grade girls, excluding early post-menarche adolescents within the first two years of menstruation. Evliyaoglu et al. emphasized critical age-dependent AMH reference ranges across puberty stages (33), yet we lacked sufficient early-puberty participants to conduct stratified age subgroup analysis. *post-hoc* power analysis revealed only moderate statistical power (0.74) driven by the small PCOS sample size (*n* = 39), generating wide AUC and diagnostic metric 95% confidence intervals. The 4.0 ng/mL optimal cut-off was derived and bootstrap-validated within this single-center cohort without external independent cohort verification; this threshold cannot be generalized to other ethnic or age adolescent populations.

### 4.1 Study limitations

1. Single-center cross-sectional school-based cohort limited exclusively to 14–18-year-old late-pubertal Chinese girls, lacking early post-menarche participants, which restricts generalizability across full adolescent age spectrums;
2. Small PCOS case sample (*n* = 39) yields moderate statistical power and wide confidence intervals for ROC diagnostic estimates; the proposed 4.0 ng/mL AMH cut-off lacks external independent cohort validation;
3. No standardized ovarian transabdominal/transvaginal ultrasound AFC or PCOM morphological data, eliminating capacity to evaluate AMH as a follicle count surrogate marker, the core research question for AMH-PCOS adolescent investigations;
4. Incorporation bias in diagnostic ROC modeling: AMH correlates with the two mandatory adolescent PCOS diagnostic phenotypes (anovulation and hyperandrogenism), potentially artificially inflating discriminatory AUC;
5. No stratified analysis by gynecological age to implement published age-specific AMH reference values across pubertal maturation stages;
6. AMH measurements performed on a single ELISA platform; divergent absolute AMH concentrations are well-documented across commercial immunoassays, limiting cross-study comparability of the 4.0 ng/mL cut-off threshold;
7. Cross-sectional design without longitudinal follow-up, unable to track dynamic AMH changes alongside pubertal HPO axis maturation or predict long-term PCOS progression risk.

## 5 Conclusion

In this population-based cohort of 14–18-year-old Chinese adolescent girls, serum AMH levels were significantly elevated in those with ovulatory dysfunction (oligo-/amenorrhea) and isolated biochemical hyperandrogenemia, and reached maximal concentrations in patients fulfilling 2023 guideline adolescent PCOS diagnostic criteria. Despite a moderate overall ROC AUC of 0.717, the bootstrap-validated optimal AMH cut-off of 4.0 ng/mL presented low diagnostic sensitivity of 51.3%, producing a high false-negative rate if deployed as a standalone screening test. Combined with major study limitations including absent ovarian ultrasound data, inherent incorporation bias in diagnostic modeling, small PCOS sample size, and alignment with official 2023 PCOS guideline recommendations against independent AMH testing for adolescent PCOS diagnosis, we conclude serum AMH functions merely as an adjunctive auxiliary laboratory biomarker rather than a primary diagnostic or population screening tool for adolescent PCOS. Large-scale multicenter prospective cohorts integrating standardized ovarian AFC ultrasound and independent external validation datasets are required to establish age-stratified, ethnicity-universal AMH diagnostic thresholds applicable across the full adolescent pubertal spectrum.

## Data Availability

The raw data supporting the conclusions of this article will be made available by the authors, without undue reservation.
